# FBG Spectrum Regeneration by Ni-Coating and High-Temperature Treatment

**DOI:** 10.3390/s22197255

**Published:** 2022-09-24

**Authors:** Carla Lupi, Cristian Vendittozzi, Erwin Ciro, Ferdinando Felli

**Affiliations:** 1 Dipartimento Ingegneria Chimica Materiali Ambiente, Sapienza Rome University, Via Eudossiana 18, 00184 Roma, Italy; 2 Campus FGA-UnB, Universidade de Brasília, Brasília 72444-240, DF, Brazil; 3 Department of Engineering Sciences, Università degli Studi Guglielmo Marconi, 00193 Rome, Italy

**Keywords:** Fiber Bragg Grating, nickel coating, electrodeposition, high-temperature applications, chirped grating, spectrum regeneration

## Abstract

FBG sensors are used in many scientific and industrial fields for assessing the structural integrity of mechanical components and in very high (above 600 °C) or very low (cryogenic) temperature applications. The main concerns with the use of such sensors in applications involving extreme temperatures are related partly to the instability of the reflected spectrum, which tends to dissolve into the noise floor, and partly to the degradation of the mechanical properties of the optical fiber, which tends to worsen the inherent brittleness. All of this raises the need for a robust nickel protective coating to ensure the grating’s integrity in high-temperature environments. In addition, the inherent brittleness of fiber-optic gratings leaves one to wonder whether it is possible to recover a broken, seemingly unusable sensor. In this way, a single-peak commercial FBG was intentionally broken in the middle of the grating length and re-spliced, inducing a strongly asymmetric chirped-like spectrum; then, a nickel coating was electrodeposited on its surface. The most important outcome achieved by this work is the regeneration of a highly distorted reflected spectrum through three thermal cycles performed from room temperature up to 500, 750, and 800 °C, respectively. After reaching a temperature of at least 700 °C, the spectrum, which has been drastically altered by splicing, becomes stable and restores its single peak shape. A further stabilization cycle carried out at 800 °C for 80 min led to an estimation of the stabilizing time of the new single-peak reflected spectrum.

## 1. Introduction

A Fiber Bragg Grating (FBG) is a passive, electromagnetic interference-insensitive device which reflects a narrow band of wavelengths (WL) when a broad band wave passes through it. FBGs are increasingly used in sensing applications, such as temperature, strain, pressure, and chemical characteristics sensors.

Those sensors have been implemented as key elements of many monitoring systems to measure the physical properties in different industrial sectors (aerospace, civil and historical infrastructure, railways, metallurgy and hydrocarbons, medical, etc.) [1,2,3], displaying notable advantages over traditional sensors such as a high resolution and accuracy of signal acquisition for static and dynamic measurements. Furthermore, multiplexing arrangements that incorporate a large number of different WL sensors into a single optical fiber greatly enhance this ability to detect slight variations in signal [2]. Moreover, the glass fiber’s inert response in acidic and electromagnetic environments, compact dimensions, and low weights have led to it being proposed as a reliable material for outdoor applications with a high stability and a low measurement attenuation, facilitating the monitoring of the surface or the bulk of structures [1,4,5]. The above-mentioned characteristics of the fiber, along with an integrated FBG setup, allow for an understanding of the ease of FBG adaptability to complex and hostile environments, giving amplified and resolved signals. 

Despite their outstanding features, a disadvantage that limits their range of use is the low resistance to very low or very high temperatures, which prevents their use in some industries where sensing systems capable of operating at extreme temperatures are required. The optical fibers used to fabricate FBGs are made of fused silica (i.e., silicon dioxide, SiO_2_) and doped with germanium silicates and other photosensitive elements to alter their refractive index. Typically, this family of materials has a very low coefficient of thermal expansion (CTE)—about 5.9 × 10^−7^/°C for a range of temperature variation between 20 °C and 400 °C, which then decreases for higher temperatures (4.8 × 10^−7^/°C, up to 900 °C), allowing the material to withstand significant thermal shocks. It has a high glass transition point at about 1200 °C, and the softening point is at about 1600 °C. 

At temperatures above 500 °C, the grating optical properties decay, which is normal for most FBG types, except those designed specifically for higher thermal exposition [6,7] or those treated by regeneration [8,9]. Recent studies explicitly aimed at the regeneration of the grating have focused their efforts on the thermal treatments, defining the thermal stabilization as the FBG regeneration process. 

In the literature, FBG regeneration has been developed by means of different techniques such as chemical composition grating (CCG), Tetrahedral FBG (TFBG) and thermal regeneration FBG (RFBG) [8]. The CCG has been characterized by multiplexing and remote interrogation properties at a high thermal stability. The influence of the doping material (e.g., Sn, B, F) on the fiber performance is significant [10,11,12]. In the case of the TFBG, a transformation from an amorphous to a tetrahedral microstructure occurs, but the optical properties and structure stability, even at high temperatures, are preserved [13]. A variation of this fiber category is nanocrystalline FBG (NFBG), which also shows a tetrahedral transformation, but more accurate thermal modifications of the fiber core favor a nano-tetrahedral phase [14]. Furthermore, one of the most used techniques is thermal regeneration. Thermal-regenerated FBG (RFBG) shows two conventional steps before its maximum performance, involving the seeding of grating and the long-lasting annealing process [15,16,17,18,19]. Moreover, hydrogen loading makes up part of the regeneration process, but one of the most remarkable features is the regeneration capability of widespread FBG types (type I-, II-, and IIa-doped and metal-coated fibers). A more detailed explanation is also discussed in Table 1.

Other attempts involving the signal stabilization have been made to restore the spectrum shape, and the numerical apodization and chirp functions have been utilized [20,21]. The former employs the well-known Sinc, Gaussian, Nuttall, and Tanh functions to enhance the grating sensitivity by increasing the spectrum ripple factor, lowering the side lobe levels, decreasing the FWHM, and removing undesirable noise [22]. In contrast, the latter numerical function is also utilized to increase the sensitivity by increasing the period, which allows the Bragg WL’s position to be linearly modified [23,24].

On the other hand, the technological challenge is to reduce thermally induced stress when gratings are exposed to extreme thermal conditions at very low (cryogenic) and very high (above 600 °C) temperatures [25,26,27]. A common-commercial polymeric coating normally covers the SiO_2_ fiber’s cladding to avoid breakups due to its brittleness. At 500 °C, the single polymeric coating on the fiber is virtually useless. Thus, several investigations have developed FBG sensing systems using high-CTE metal coatings as thermal protective barriers [28,29]. Such protective coatings for high-temperature applications (aircraft engines, oil wells, combustion processes, chemical and metallurgical industries) require, from the coating processing, that the sensor maintains the physical integrity, high sensitivity, and stability of the fiber. Hence, physical vapor deposition (PVD) [30,31], chemical vapor deposition (CVD), casting, electroless [32], chemical deposition, and electrodeposition [27,33,34] have been considered to perform the metal coating on the FBG sensor. In particular, electrodeposition is the most promising technique for producing thick metal coatings on conductive surfaces at room temperature (RT), controlling dimensional, morphological, and purity features via operative conditions optimization [27].

The proper selection of deposition techniques, operating conditions, and metal types for primary and multilayer (secondary) coatings on the FBG sensor plays a crucial role in improving sensitivity to thermal or mechanical variations. In this way, the non-conductive silica cladding demands sputtered primary layers (Au, Al, Cu, Zn, etc.) to make the FBG surface conductive and ensure the metal electrodeposition [22]. In contrast, the properties of the metal coating are decisive in enhancing the FBG performance [29,35].

The first approach to enhance the grating sensitivity between the coating and fiber proposes the deposition of metal coatings with a very high coefficient of thermal expansion (CTE) and Young’s modulus (E) compared to those of silica fiber. However, it can be subjected to critical thermal stresses, producing both the transmitted spectra distortion and destructive coating detachment from the fiber. The influence of thickness on the thermal resistance also indicates that thin coatings commonly have a lower inertia, quickly following temperature variations, whereas thick layers amplify the grating’s response. To understand the thickness effect on FBG performance, some studies have been carried out on the modeling and experimental optimization of operative conditions [25,36], indicating that both the metal and thickness strongly affect the grating sensitivity. Moreover, the fiber metal coating suffers from compressive strains when temperatures decrease, while it elongates if temperatures increase. In this context, an adequate thickness should be chosen depending on the metal used for the coating and its characteristics, suggesting optimization assessments, and considering that: (i) if the metal has high elastic properties (E), it shows no deformation at a low thickness, whereas (ii) a significant deformation is observed at a higher thickness with metals with low elastic properties. Finally, (iii) high-CTE metals show higher deformations demanding a higher thickness.

Metal coatings of high melting temperatures should be chosen to facilitate strain transfer and reduce thermal stress without being close to or exceeding the melting point of the fiber. Some metals with low melting points, such as In, Pb, and Sn, appear to perform better under cryogenic conditions than at high temperatures [37,38], whereas Ag and Au, which are hardly affordable, can withstand temperatures of up to 962 and 1064 °C, respectively. Otherwise, low-cost metals such as Cu and Ni have been thought to protect fibers at high temperatures due to their melting points being above 1000 °C. A recent work has been carried out to develop a double-metal layer system made of a Cu and Ni coating on FBG for high-temperature applications. The best results suggest a real improvement of the double-coated FBG working correctly up to 700 °C without the interdiffusion phenomenon between the binary layer metallic coatings, but a critical effect on the coating was observed as the temperature increased, causing the progressive formation of the Kirkendall voids [34].

In the past, the temperature influence on grating sensitivity has been studied in Ni-coated FBGs prepared via electroless [32,39]. However, electrocoated-Ni FBG sensors at high temperatures, used as a protective coating, have been rarely considered in the literature. Due to nickel’s capacity to bear high temperatures, the glass fiber’s performance as a thermal protective coating could be improved.

Therefore, the primary purpose of the present work is to evaluate the possibility of regenerating the reflected spectrum of re-spliced gratings through a series of thermal cycles performed at high temperatures on a Ni-coated sample to recover the single-peak original shape. Such a nickel coating was deposited on a sensor that was intentionally broken in the middle of the grating length and then re-spliced, inducing a strongly asymmetric chirped-like spectrum. 

## 2. Materials and Methods

**Figure 1:**
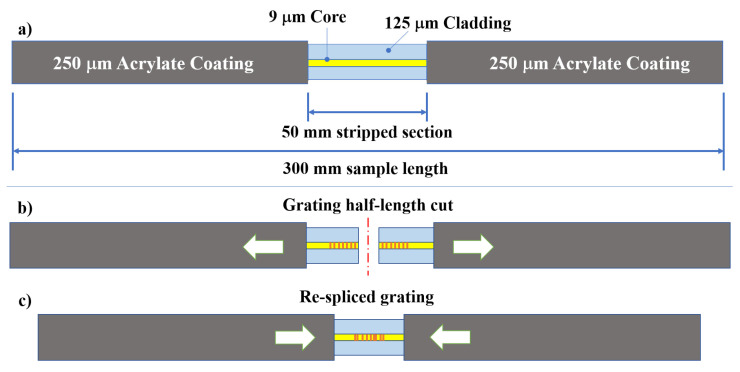
Specimens’ configuration. (**a**) The so-called “dummy sample” consists of a 9/125 bare fiber segment; (**b**) the sample carrying the Bragg grating was first cut at its half-length and then (**c**) re-spliced.

The present work was carried out using a germanium-doped single-mode 9/125 optical fiber (i.e., a 9 µm core and 125 µm cladding) externally protected by 250 µm of acrylate coating to allow it to be handled. The test specimens were taken from the same fiber by cutting 300 mm segments. From the latter, the coating of the central part was removed (stripped) for a length of 50 mm. The specimen configuration is shown in Figure 1.

**Figure 2:**
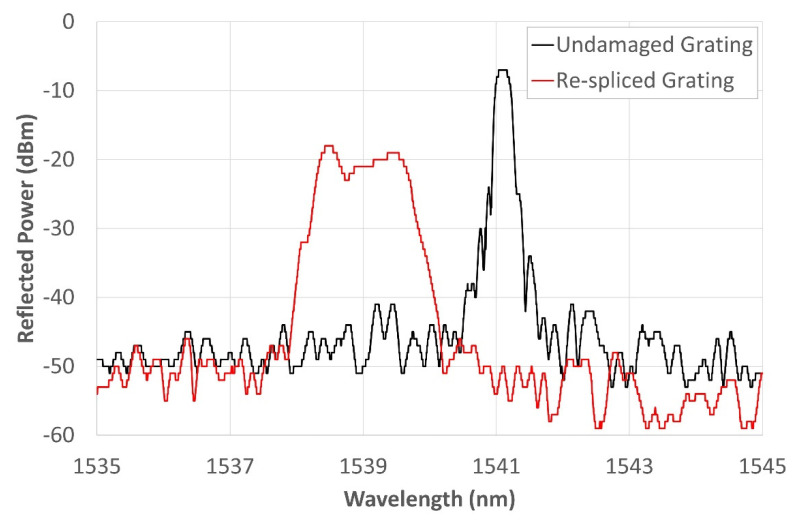
Comparison between the spectra of the same grating before and after intentional breaking and subsequent re-splicing.

The dummy samples were used to optimize the electrodeposition process that was then used to coat the rejointed sensor in order to submit it to high-temperature heat treatment (heating and cooling) in an attempt to regenerate it. Four more samples, with the same configuration presented in Figure 1 but containing a 20 mm-long grating, were used in the test campaign. Those gratings were cut in half (Figure 1b) using a Miller fiber optic stripper. The two ends were trimmed with a Fujikura optical fiber cleaver HS 30 to make sure that both ends were perfectly orthogonal to the longitudinal axis of the fiber in order to ensure optimal welding. Then, the fibers were spliced again using a Sumitomo type 46 fusion splicer (Figure 1c). The gratings spectra were logged by the FS22 SI Fibersensing interrogator, providing a resolution of less than 0.5 pm. The interrogator was used to monitor FBGs during the whole electrodeposition and thermal cycles campaign. The cutting of the gratings and the subsequent splicing caused significant deformation of the gratings’ spectra. An example of one of the new re-spliced spectra and its original spectrum is shown in Figure 2, where the black curve represents the reflected spectrum of the original grating and the red curve represents the highly distorted spectrum generated by the splicing. Both spectra were taken at RT (20–22 °C). The original spectrum has its central wavelength (CWL) localized at 1541.01 nm (easily identifiable), with a full width at half-maximum (FWHM) of about 0.555 nm. 

As a reference for measuring the amplitude of these spectra, the base of the two main “bells” was identified at −50 dB, which is the amplitude where the noise floor of the two spectra is concentrated. The original spectrum has its peak at −7 dB, so the reflected power amplitude is calculated as 43 dBm. The new spectrum decreases in amplitude by about 13 dBm, while the FWHM increases by about 1.95 nm. The new spectrum no longer has a clearly identifiable CWL. It is instead a rather asymmetrical chirped grating, with the two edge peaks located at 1538.44 nm (−18 dBm) and 1539.505 nm (−19 dBm), respectively, and a central depression (corresponding to a local minimum) at 1538.785 nm (−23 dBm). After the splicing, the spectrum decreases in amplitude, broadens, and shifts stably to the left. The spectrum of this grating is used in this paper to show the whole process and the achieved results; the other gratings showed comparable results. 

**Figure 3:**
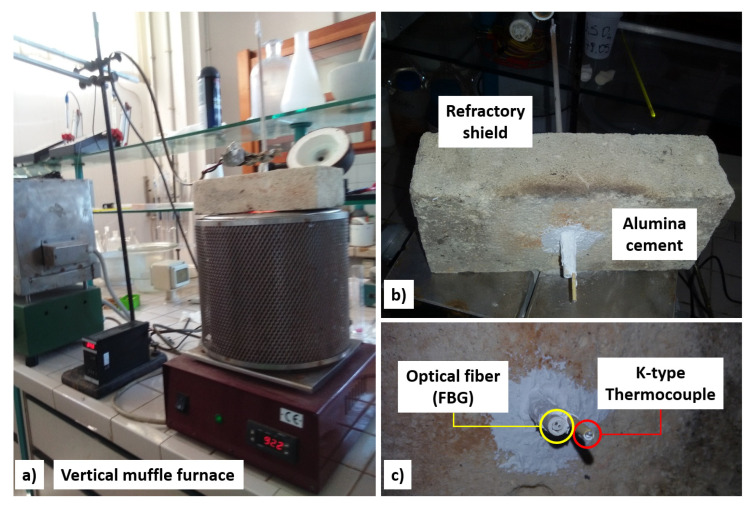
(**a**) Muffle furnace used for high-temperature thermal cycling. (**b**) Refractory feedthrough. (**c**) Detail of the feedthrough consisting of two alumina tubes; the one on the left is a double coaxial tube for passing the fiber optic (FBG), and the one on the right is for passing a K-type thermocouple. The system was sealed with alumina cement.

All thermal cycles were carried out using a vertical muffle furnace (Figure 3a). The top of the furnace was closed by means of a refractory shield (Figure 3b) of a size larger than the furnace aperture. The SiO_2_ fiber carrying the testing grating entered the furnace through a feedthrough consisting of a double coaxial alumina cannula sealed with alumina cement (Figure 3c). Through a second tube, parallel to the first, a K-type thermocouple was fed in to read the actual temperature in the very proximity of the grating, independent of the temperature displayed by the muffle thermocouple. The grating was kept in thermal and mechanical equilibrium at room temperature for 15 h to check for any environmental interference that might have interfered with the test. The sensor remained in the same position for the entire duration of the tests.

### 2.1. Preparation of the Ni-Coated Sensor

**Figure 4:**
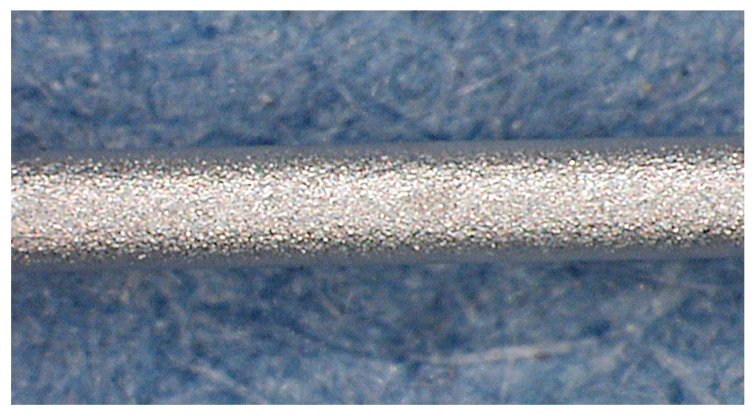
External appearance of Ni-electrodeposited coating.

**Figure 5:**
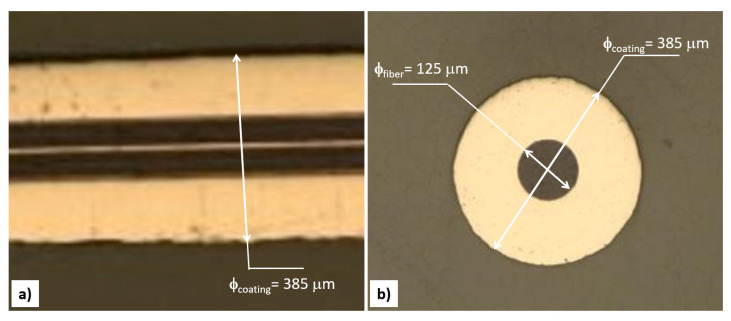
The appearance of the Ni-coated fiber at the (**a**) longitudinal and (**b**) cross section. The figures show the diameter of the optical fiber (125 mm) and the diameter of the coating (385 mm); the thickness of the coating (about 130 mm) can be deduced from these dimensions.

One of the main issues in performing a metal coating is facilitating the complete charge flow during the whole circuit. The non-conductive surface of the glass fiber requires a surface treatment to ensure the electrical circuit closure and therefore the ability to deposit the Nickel ions. To make the cylindrical surface electrically conductive, the 50 mm stripped segments of the fiber were gilded using an EDWARDS sputter coating, model S150B. The electrodeposition was then carried out by immersing the gilded segment into the cylindrical glass cell, where it worked with a cylindrical lead (Pb) as the cathode and anode, respectively. The fiber was placed along the longitudinal axis of the electrodeposition cell to maintain the uniformity of the thickness coating with respect to the diameter. As mentioned, such a fiber arrangement was adopted to obtain the regular thickness of the coating but mainly to avoid the heterogeneous growth of layers due to anisotropic radial stresses that negatively create undesirable behaviors over the signal, altering the grating spectrum. The electrolyte containing the nickel ions was prepared by dissolving 40g/L NiSO_4_·6H_2_O and 20 g/L H_3_BO_3_ from Carlo Erba pure chemical reagents. Once the electrodes were immersed in a 500 mLelectrolytic bath, Ni-coated FBG sensors were obtained at pH 4.8 for a process duration of 180 min. A potentiostat/galvanostat Amel Instrument (model 2053) was used to carry out the nickel deposition at a 50 A/m^2^ current density (CD) and RT. Dummy samples were observed using an optical Zeiss microscope. The aspect of the Ni-coated fibers is shown in Figure 4 and Figure 5. The nickel coating on the fiber displayed a regular and compact aspect in cross and longitudinal sections (in Figure 5a,b, respectively). The selected experimental conditions achieved a well-distributed Nickel coating, of a thickness of about 130 mm, without apparent porosity, cracks, or internal defects, as shown in Figure 4 and Figure 5. 

### 2.2. Thermal Recovery and Cycling Treatment

The hydrogen evolution reaction (HER), characterized by the formation of bubbles on the cathodic surface (Ni cathode), was apparently bypassed by using low CD and maintaining the constancy of the electrolyte pH. Nevertheless, HER causes the hydrogen absorption that usually occurs on the metal deposit during the ED [40,41]. As is well known, Ni is a good hydrogen electro-catalyst; thus, thermal conditioning (recovery process) is required to facilitate the dehydrogenation process and eliminate internal defects (stress or porosity). Therefore, the Ni coating recovery was carried out at a rate of 2 °C/min, from RT up to 170 °C. The operative parameters used during the recovery stage to eliminate the hydrogen content and internal stresses of the electro-coated FBG can be seen in Table 2.

The nickel-coated sensor was then thermally cycled, with steps of heating from RT to 500, 750, and 800 °C, followed by cooling back to RT (Table 2).

## 3. Results and Discussion

### 3.1. Analysis of the Spectrum during Gilding, Electrodeposition, and Thermal Recovery

The reflected spectrum of the re-spliced grating was observed throughout the test campaign, starting from the gilding and electrodeposition phases of the nickel coating. 

As already shown in Figure 2, the new spectrum, resembling that of a highly asymmetric chirped sensor, is characterized by the presence of two spaced peaks. This shape is due to the re-splicing of the grating carried out approximately in the center of it. The splice reduced the amplitude of the spectrum and generated a second peak, probably due to an alteration of the SiO_2_ structure generated in the splice zone. This condition, although not relevant to determining the effectiveness of the metal coating, provided an opportunity to test whether the original sensor could be regenerated through heat treatments. 

**Figure 6:**
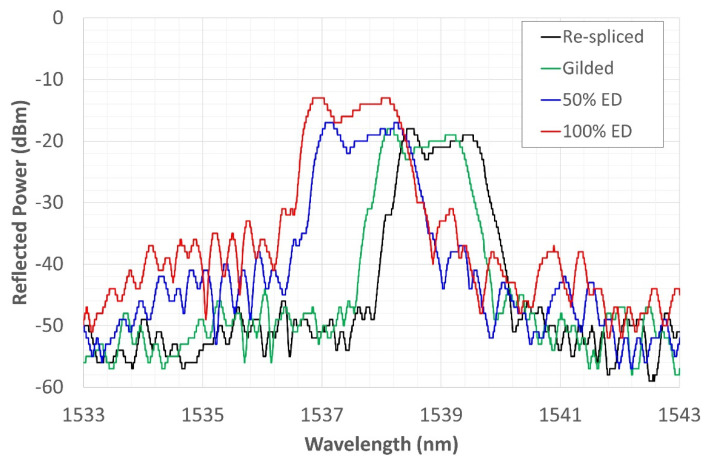
Grating reflected spectrum change during electrodeposition compared with the initial conditions of the re-spliced fiber.

Figure 6 shows the shifting of the sensor signal to the left during the gilding phase (green solid line), and later, in the electrodeposition phase, a shift occurred, again toward shorter WLs; while the reflection power tends to increase, the amplitude is reduced due to a slight increase in the noise floor. This condition can be explained by the onset of compressive stresses due to the formation of the gold and, therefore, nickel coating. Furthermore, during the nickel electrodeposition, there may also be a minimum effect due to the hydrogen, which, by placing itself in an interstitial position in the nickel lattice, increases the coating tension state. The change in the reflected spectrum in Figure 6 demonstrates good electrodeposition quality since, by not further altering the shape of the new spectrum, it preserves the functionality of the grating. This condition of shape retention and mere spectrum translation is indicative of the uniformity of the deposited Nickel layer; otherwise, there would have been points at which the sensor would have undergone expansions and others at which there would have been compressions, giving an undesired shape to the spectrum.

The temperature variation is responsible for the thermal expansion and/or compression of the sensor grating and for the refractive index change, effects that could cause a consistent variation in the reflected spectrum. Thus, the temperature was kept constant using a 20 °C thermostatic bath and carefully controlled during electrodeposition to ensure that the only stress the sensor underwent during electrodeposition was the mechanical one, related to the nickel coating on its surface.

**Figure 7:**
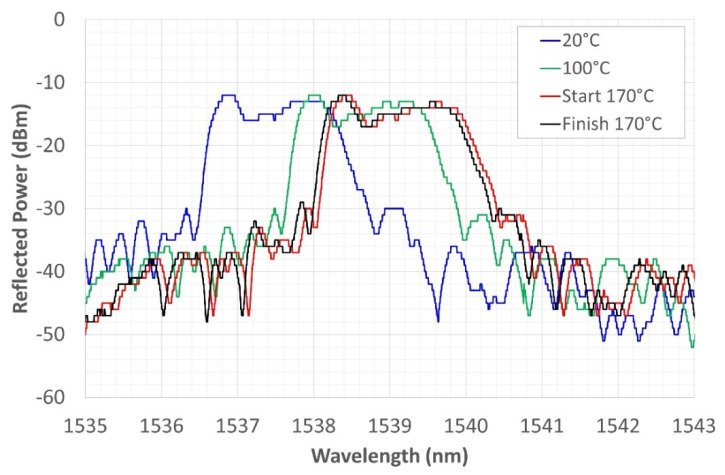
Grating reflected spectrum change during the recovery at 170 °C.

The Ni-coated FBG was subjected to a recovery treatment at 170 °C to eliminate hydrogen absorbed during Ni electrodeposition. As was expected, the spectrum progressively translated toward higher WLs (Figure 7) as an effect of the temperature increase with consequent stress release. However, the shift is negligible between the start and finish of the recovery treatment (the red and the black line on the graph in Figure 7, respectively), indicating that the stress induced by hydrogen desorption is very small.

### 3.2. High-Thermal Cycling 

High-temperature thermal tests were performed to stabilize the grating, prevent the occurrence of critical defects in the deposit and in the spliced grating, and investigate the aptitude of the Ni-coating to act as a grating protection, along with its functionality in such critical conditions. 

**Figure 8:**
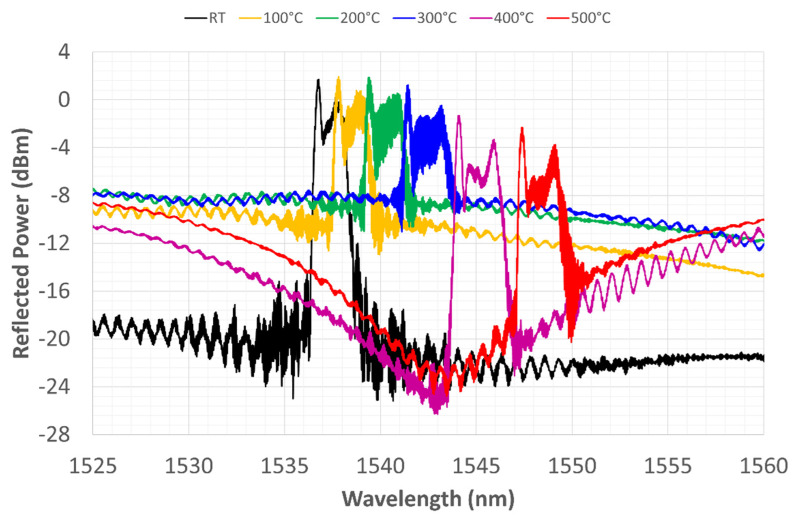
First thermal cycle, grating reflected spectrum change during heating from RT to 500 °C.

In the first thermal cycle, the sensor was heated up to 500 °C and then left to cool down to RT in the switched-off furnace. Figure 8 shows the WL shift during the heating phase. The black line represents the spectrum at RT, and the red line represents the spectrum at 500 °C. All the graphs of the heating phase should be read from left to right, following the translation of the spectrum that shifts to the right due to the increase in temperature. The transformation of the spectrum with temperature depends partly on the dependence of the thermal-optic coefficient on temperature and partly on the CTE of the involved materials. According to [42], the thermal-optic coefficient decreases parabolically up to 400 °C and then increases again, following the same second-degree polynomial approximation, while the refractive index tends to increase linearly over the same temperature range. The diffraction coefficient remains almost constant up to 700 °C and then falls in the range up to 800 °C. 

The CTE of SiO_2_ although it is at least two orders of magnitude lower than that of nickel, increases between 0 and 300 °C and then decreases to 900 °C. Thus, the expansion of nickel drags the fiber throughout the considered temperature range. During expansion, residual stresses that have been stored between the core and cladding during electrodeposition tend to diffuse, causing the relaxation of the fiber’s structure and, consequently, of the grating photo-written in it. During heating, the grating’s reflection power (its maximum value at the peaks) remains unchanged up to 200 °C. The amplitude, on the other hand, seems to decrease, inversely proportional to the increase in temperature. This is only an apparent phenomenon due to the rise of the noise floor, which fluctuates as if carried by a sinusoidal driving signal. The distance between the two peaks at the ends increases with their height, as if the split-peak shape depended on increasing temperature.

The reflected power decreases gradually but unevenly as the temperature increases, (Figure 8). Moreover, there is no obvious proportionality between temperature increase and WL displacement. Seemingly, only the shift in CWL grows proportionally with increasing temperature, while Figure 8 shows that the rightward shift becomes more evident at higher temperatures, where the coating expands more because the conductivity and CTE of Ni are both higher [43,44]. The split-peak configuration shown at RT is still present in this temperature range. 

**Figure 9:**
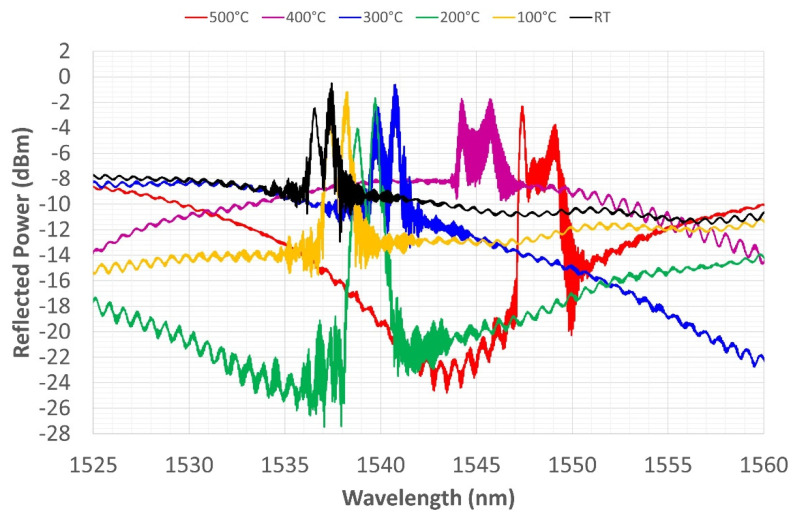
First thermal cycle, grating reflected spectrum change during cooling from 500 °C to RT.

At 500°C, the furnace is turned off, keeping it closed so that it slowly returns to RT. All the graphs of the cooling phase should be read from right to left, again following the spectrum shift toward lower WLs, consistent with the lowering of temperature. In Figure 9, the spectrum reflection power trend increases again and again. 

The reflected power trend changes as already described in this section, while the amplitude undergoes apparently inconsistent variations, but once again, its variation is related to the sinusoidal driving oscillation of the noise floor. Figure 9 shows another interesting phenomenon: while, during heating, the spectrum peak with the highest reflection power was the left peak (corresponding to the smaller WL), during cooling, the highest peak (i.e., the WL with the highest reflection power) becomes the right peak.

**Figure 10:**
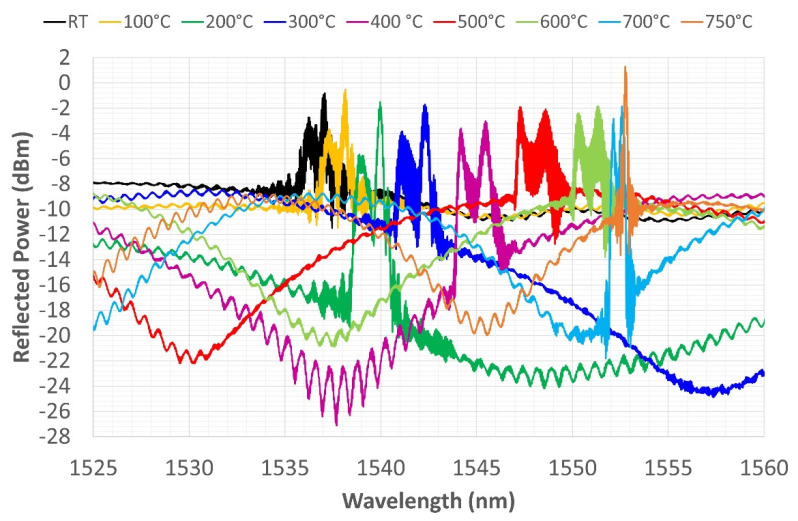
Second thermal cycle—grating reflected spectrum change during heating from RT to 750 °C.

**Figure 11:**
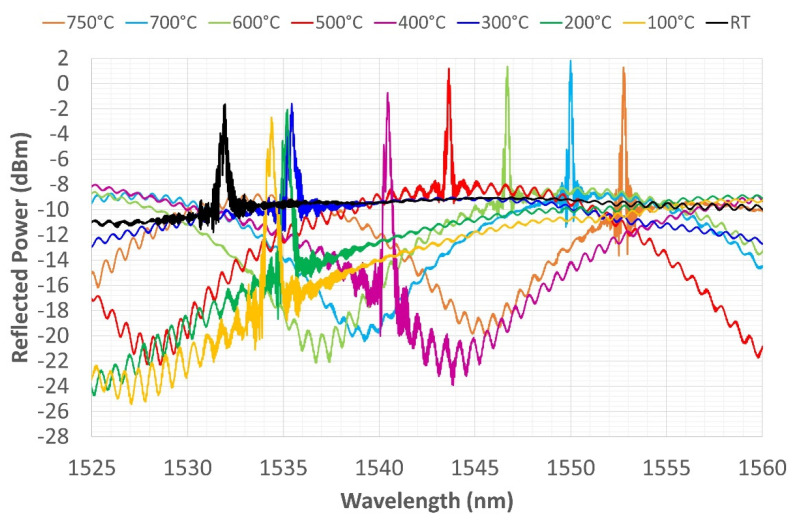
Second thermal cycle—grating reflected spectrum change during heating from 750 °C to RT.

In the second thermal cycle, the furnace is heated up to 750 °C (Figure 10) and then cooled down to RT (Figure 11). A relevant phenomenon was the disappearance of the spectrum split-peak, which occurred during the heating phase at 750 °C. Losing one of the two peaks reduces the FWHM of the spectrum by about 76%. The disappearance of the split-peak could be explained by a reorganization of the germanium fringes, constituting the sensor, induced by high temperatures. The new spectrum at the end of the second cycle clearly highlights the disappearance of one of the two peaks from the reflected grating spectrum. The spectrum maintains the new shape up to RT, but the FWHM tends to increase slightly; after 400 °C, a second peak reappears, which is less pronounced than it is in the previous shape (Figure 11).

**Figure 12:**
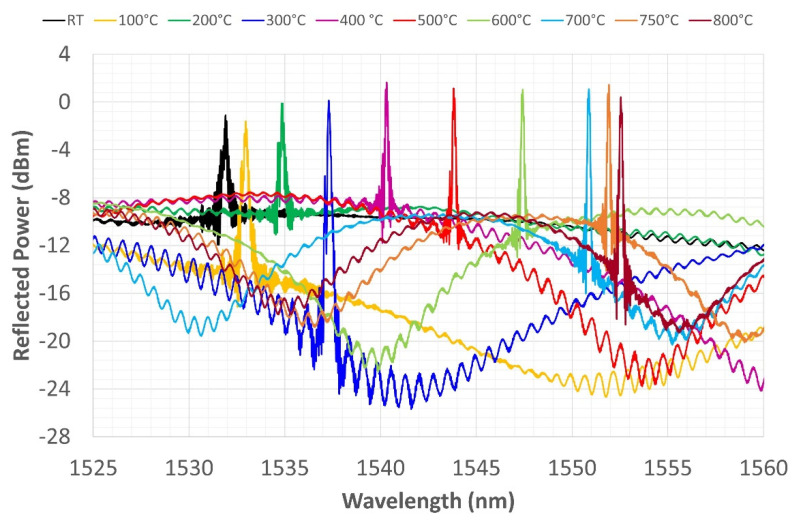
Third thermal cycle—grating reflected spectrum change during heating from RT to 800 °C.

**Figure 13:**
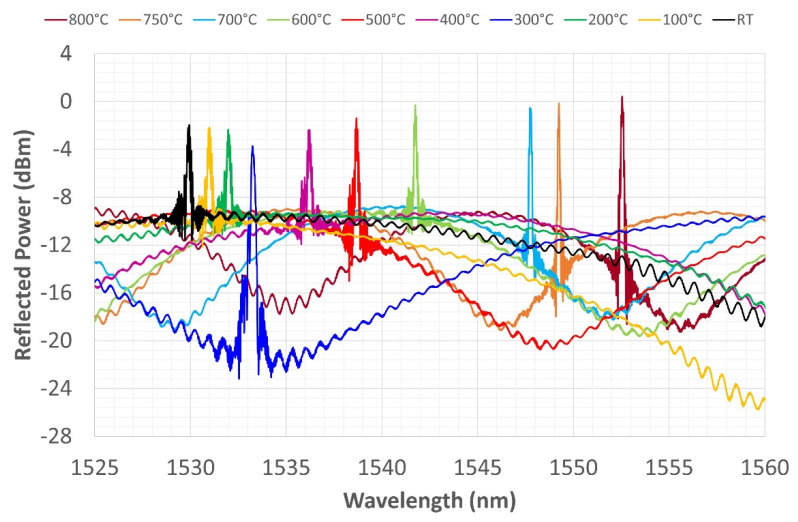
Third thermal cycle—grating reflected spectrum change during cooling from 800 °C to RT.

The last thermal cycle reached a temperature of 800 °C. Figure 12 and Figure 13 show very similar behavior to that shown in relation to the previous cycles. During heating (Figure 12), the spectrum shows two peaks up to 300 °C, which then disappear from 400 to 800 °C. The spectrum retains only one peak during cooling, the shape becomes more tapered, and a more pronounced side lobe reappears between 500 and 400 °C. The CWL is always well defined at the end of the third cycle, even though the side lobe hints occasionally return at lower temperatures. The reflection power remains almost constant during the cycle, while the amplitude, again, fluctuates depending on the sinusoidal variation of the noise floor driving signal.

### 3.3. Thermal Cycle for CWL Stabilization

**Figure 14:**
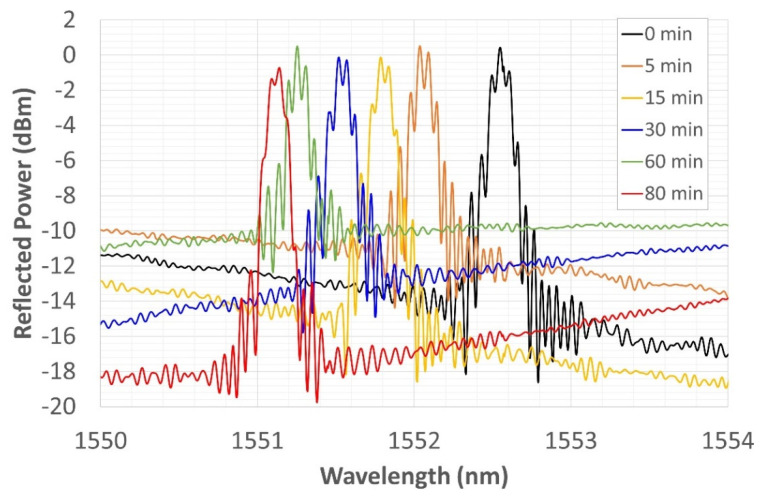
Thermal stabilization cycle at 800 °C for CWL stabilization.

The three thermal cycles led to the grating regeneration, which regained its conventional shape with a central peak. With the aim of stabilizing the CWL, the sample was submitted to a further thermal cycle. In this case, it was a stabilization of 80 min at a temperature of 800 °C. Figure 14 shows the test result, logging the spectrum as a function of stabilization time. The acquisition system that was used during the test campaign does not allow for an automatic saving of the reflected spectrum. Therefore, the test was carried out by manually saving the spectrum after a certain time interval; the results are summarized in Table 3. During the cycle, the spectrum that had been taken to 800 °C from RT made the expected rightward shift, reaching the same WL recorded in the third thermal cycle (i.e., 1552.545 nm at 800 °C), and then began to shift leftward. 

The shift was most relevant in the first 5 min (when it reached 1552.035 nm). The effect was reduced after 15 min. The spectra trended to be increasingly close, concentrating on a given WL. The cycle was interrupted after 80 min, with the CWL at 1551.145 nm. The CWL is not yet stable; its trend to a certain wavelength leads us to think that a longer stabilizing time interval is needed. 

**Figure 15:**
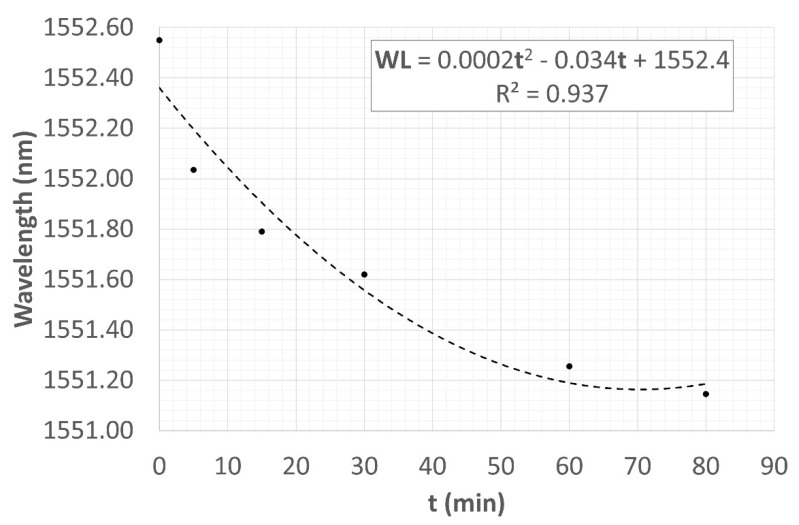
CWL stabilization trend.

Figure 15 shows the second-degree polynomial trend curve of the stabilizing CWL. This curve was obtained from the experimental data collected in the second column of Table 3, while the third column represents the estimated trend calculated through the trend curve shown in Figure 15. This curve, which was drafted with the aim of identifying a plausible stable WL for this temperature value, reaches a minimum after 85 min at CWL_est_ = 1550.9550 nm and then increases again. 

Although the fitting curve changes its sign after 85 min, such behavior would have to be rejected considering that the spectrum is at its minimum energetic point, so a shift increase should need a further energetic input.

## 4. Conclusions

The present work had two main objectives: (i) producing an electrodeposited Ni-coating for mechanically and thermally protecting the FBG and (ii) evaluating if high-temperature thermal cycles performed on it could help to regenerate its highly asymmetric reflected spectrum, which was generated by cutting the grating at half its length and then re-splicing it. The electrodeposition, performed at RT, generated a compressive state on the grating, inducing a rightward shift of the CWL by about 1.5 nm; the reflected power increased by about 14% and the FWHM increased by 19%, while the chirped-like shape was maintained. The subsequent recovery, performed at 170 °C, shows that the hydrogen desorption effect was negligible, as the spectrum shape and main parameters remained almost unchanged. During heating from RT to 170 °C, the reflected power and FWHM showed no changes, while, predictably, the WL shifted to the right.

The three thermal cycles that were carried out at higher temperatures—up to 500 °C, up to 750 °C, and up to 800 °C, respectively—demonstrated how, if adequately protected by the nickel coating, the grating is able to respond effectively, even at temperatures that exceed conventional operating limits. Moreover, it reveals how the same cycles can provide the regeneration of the reflected spectrum, intended, on this occasion, as the regeneration of the single central peak shape, even for a spectrum that was severely deformed by a re-splicing that occurred at the grating length. The choice to re-splice an FBG, which has been cut along the grating itself, was made in order to generate, in a completely random manner, a new spectrum. It was the authors’ intention that the spectrum that is to be regenerated will not have a predefined and predictable structure, allowing for a more general treatment of the regeneration principle that can, in theory, be proposed on the spectra of any shape.

The results show a coherent response to temperature changes of up to 700 °C. At 750 °C, the spectrum went into a severe transformation caused by a partial deletion of the reflected spectrum itself due to the dopant diffusion and, probably, to the subsequent growth of a new spectral band; furthermore, a combination with the SiO_2_ Optical Fiber residual stress relaxation led to an alteration of the fundamental parameters that characterize the shape of the reflected spectrum. A further stabilization cycle (a kind of annealing of the Ni coating) stabilizes the new reflected spectrum. An attempt was made to predict the stabilization point using a second-degree polynomial fit. 

The outcomes demonstrate that the electrodeposited Ni-coating allows the grating to survive the high temperatures chosen for the regeneration cycles. Furthermore, the work reveals that those thermal treatments are able to regenerate a reflected spectrum after the grating breakage and its subsequent splicing, returning a conventional shape to a spectrum that had deformed in an uncontrolled manner.

## Figures and Tables

**Table 1:** Summary of regeneration techniques for high-temperature FBG applications.

Technique	Brief Explanation	Details	Ref.
Chemical composition grating (CCG)	Hydrogen-loaded FBGs are written in doped compounds capable of achieving thermal stabilization at temperatures above 1000 °C, while optical properties are altered (i.e., the presence of large-scale multiplexing and remote interrogation can occur).	- Tin-doped FBG performed up to 826.7 °C [10]. - Boron-doped highly silica fiber worked until 1295 °C [11]. - Fluorine-doped silica fiber with a thermal operation ranging from 1000 to 1200 °C [12].	[10,11,12]
Tetrahedral FBG (TFBG)	Fibers are transformed from amorphous to tetrahedral-dominated microstructures. The modified fiber shows similar optical properties to conventional FBGs, but the tetrahedral microstructure shows a high thermal tolerance without losing grating and structural stability.	- Co-doped Type-I FBG or Type-II FBGs annealed at 1100 °C. They can work up to 1200 °C [13]. - NFBG [14].	[13,14]
Thermal-regenerated FBG (RFBG)	The thermal RFBG entails two typical steps: seed grating followed by isothermal or long-lasting treatment (annealing). A hydrogen loading is the first step prior to performing the seeding. The main scope of this technique is to thermally treat doped or conventional FBGs to stabilize the grating and work for high-temperature applications.	- Conventional FBG type I, type II/type IIa gratings heated up to 800 °C [15,17,18,19]. - Novel Er-YZCAPS (SiO_2_-Al_2_O_3_-ZrO_2_-Y_2_O_3_-CaO-P_2_O_5_-Er_2_O_3_) fiber worked up to 1400 °C and regenerated by a conventional annealing treatment [16].	[15,16,17,18,19]

**Table 2:** Operative parameters for the thermal recovery and cycling treatment.

Thermal Stage	Temperature (°C)	Heating Conditions	Propose
Recovery	170	2 °C/min	Dehydrogenation and stress elimination
I Thermal cycle			Evaluation of high-temperature effect on coating
Heating	20–500	2 °C/min	
Cooling	500–20		
II Thermal cycle			Evaluation of high-temperature effect on coating
Heating	20–750	2 °C/min	
Cooling	750–20		
III Thermal cycle			Evaluation of high-temperature effect on coating
Heating	20–800	2 °C/min	
Cooling	800–20		

**Table 3:** Thermal stabilization cycle at 800 °C; experimental data.

t (Min)	WL_Exp_ (nm)	WL_Est_ (nm)
0	1552.550	1552.400
5	1552.035	1552.235
15	1551.790	1551.935
30	1551.620	1551.560
60	1551.255	1551.080
80	1551.145	1550.960
81	-	1550.958
82	-	1550.957
83	-	1550.956
84	-	1550.955
85	-	1550.955
86	-	1550.955
87	-	1550.956
88	-	1550.957
89	-	1550.958
90	-	1550.960

## Data Availability

Not applicable.
